# Quantification of TFF3 expression from a non-endoscopic device predicts clinically relevant Barrett's oesophagus by machine learning

**DOI:** 10.1016/j.ebiom.2022.104160

**Published:** 2022-07-15

**Authors:** Adam G. Berman, W. Keith Tan, Maria O'Donovan, Florian Markowetz, Rebecca C. Fitzgerald

**Affiliations:** a Cancer Research UK Cambridge Institute, University of Cambridge, Cambridge, UK; b Early Cancer Institute, Department of Oncology, Hutchison Building, University of Cambridge, Cambridge, UK; c Department of Gastroenterology, Addenbrooke's Hospital, Cambridge University NHS Foundation Trust, Cambridge UK; d Department of Histopathology, Addenbrookes Hospital, Cambridge University NHS Foundation Trust, Cambridge, UK; e Cyted Ltd, Cambridge, UK

**Keywords:** Barrett's oesophagus, Trefoil-factor 3, Intestinal metaplasia, Cytosponge, Non-endoscopic devices, Machine learning

## Abstract

**Background:**

Intestinal metaplasia (IM) is pre-neoplastic with variable cancer risk. Cytosponge-TFF3 test can detect IM. We aimed to 1) assess whether quantitative TFF3 scores can distinguish clinically relevant Barrett's oesophagus (BO) (C≥1 or M≥3) from focal IM pathologies (C<1, M<3 or IM of gastro-oesophageal junction); 2) whether TFF3 counts can be automated to inform clinical practice.

**Methods:**

Patients from the Barett's oEsophagus Screening Trial 2 (BEST2) case-control and BEST3 randomised trials were used. For aim 1, TFF3-positive glands were scored manually and correlated with clinical diagnosis. For aim 2, machine learning approach was used to obtain TFF3 count and logistic regression with cross-validation was trained on the BEST2 dataset (*n* = 529) and tested in the BEST3 dataset (*n* = 158).

**Findings:**

Patients with clinically relevant BO had higher mean TFF3 gland count compared to focal IM pathologies (mean difference 4.14; 95% confidence interval, CI 2.76-5.52, *p* *<* 0.001). The mean class-balanced validation accuracy was 0.84 (95% CI 0.77-0.90), and precision of 0.95 (95% CI 0.87-1.00) for detecting clinically relevant BO. Applying this model on BEST3 showed precision of 0.91 (95% CI 0.85-0.97) for focal IM pathologies with a class-balanced accuracy of 0.77 (95% CI 0.69-0.84). Using this model, 55% of patients (87/158) in BEST3 would fall below the threshold for clinically relevant BO and could avoid gastroscopy, while only missing 5.1% of patients (8/158).

**Interpretation:**

Automated Cytosponge-TFF3 gland quantification may enable thresholds to be set to trigger confirmatory gastroscopy to minimize overdiagnosis of focal IM pathologies with very low cancer-associated risk.

**Funding:**

Cancer Research UK (12088/16893 and C14478/A21047).

**Research in context**

**Evidence before this study**

We searched PubMed from database inception to 30^th^ March 2022 with the MeSH terms ‘Trefoil Factor-3’ (TFF3) and Barrett's oesophagus and ‘quantification[tw]’ and to review the status of the scientific literature and found literature on the use of TFF3 quantification in human saliva, but no literature on its use in Barrett's oesophagus (BO). Intestinal metaplasia (IM) is a hallmark of BO and compared with focal IM of the gastro-oesophageal junction, the more extensive IM in BO or the gastric mucosa is thought to increase the risk for cancer. Detection of IM using non-endoscopic methods such as the Cytosponge coupled with the biomarker for IM, TFF3 may be used as a screening test for BO or early OAC. In clinical trials (BEST1, BEST2, BEST3) to date, all TFF3-positive patients have had a gastroscopy. However, patients who are TFF3 positive may have focal IM pathologies with very low cancer risk and gastroscopy could be obviated.

**Added value of this study**

A quantitative assessment of TFF3 correlates with the length of the BO segment. An automated prediction model can be used to accurately quantify the extent of IM from Cytosponge specimens without requiring the pathologist to perform a manual count and this model had a 90% precision for identifying focal IM pathologies.

**Implications of all the available evidence**

When applying the Cytosponge in a screening population, these results will enable clinicians to categorise TFF3 positive participants into those with 1) clinically relevant long segment BO who will require gastroscopy; or 2) focal IM pathologies such as IM of the GOJ or short segment BO that may not be clinically significant and reassurance or a follow-up Cytosponge in 2-3 years may be sufficient.

Alt-text: Unlabelled box

## Introduction

Barrett's oesophagus (BO) predisposes to oesophageal adenocarcinoma (OAC), a tumour with an abysmal 5-year survival of *<*20%.^1^^,^^2^ The poor survival from OAC is related to late presentation such that 40% of patients with OAC have distant metastases at presentation.^3^ Hence, there is imperative to find ways to improve the early detection of OAC and screening is a consideration.

**Figure 1:**
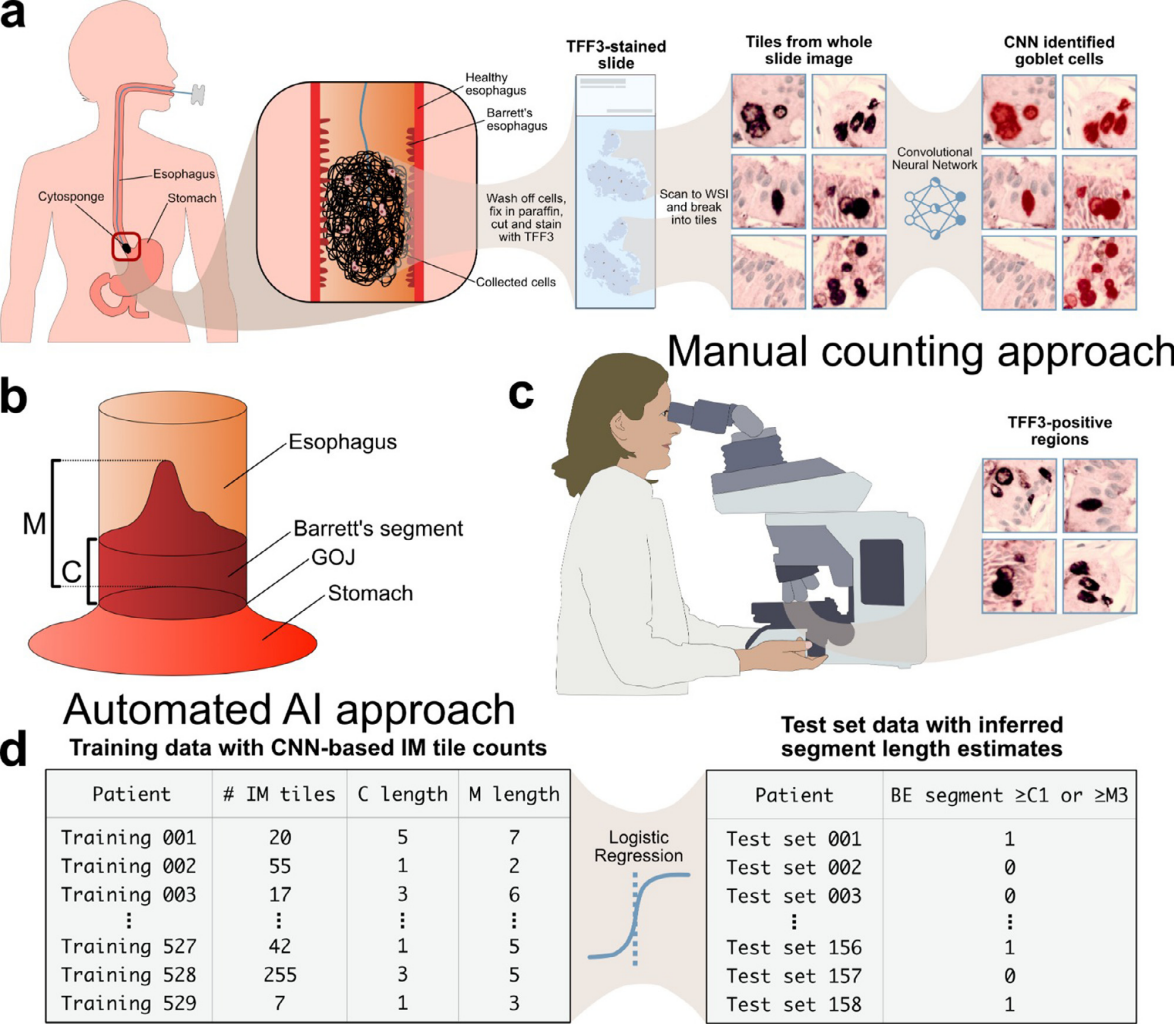
**Overview of Cytosponge-TFF3 preparation, Prague classification, and computational pipeline. (a)** The Cytosponge collect cells from the entire oesophagus, including BO epithelium, if present. Slides are generated for TFF3 immunohistochemistry and then scanned into whole-slide images (WSIs), which can then be broken up into thousands of smaller images (“tiles”). These tiles are labelled by a pathologist for the presence of TFF3 positive goblet cells indicating IM, and a convolutional neural network is trained to perform this classification task from these labels. **(b)** A diagram showing how the C and M lengths of the Prague classification criteria for segment length measurements. C (circumferential) denotes the distance from the proximal margin of the gastric folds to the proximal margin of the circumferential BO segment, and M (maximum) describes the distance to the most proximal extent of the BO segment. **(c)** Expert pathologists manually counted TFF3 positive gland groups in Cytosponge slides for correlation with the segment length. **(d)** The number of 'tiles’ classified as showing IM among those stained positive for TFF3 on slides in the training set was used to train a logistic regression model to predict the BO segment length. BO segment lengths (C & M) are in centimetres.

There has been debate as to whether the presence of intestinal metaplasia (IM) is required to diagnose BO. The British Society of Gastroenterology (BSG) stipulates that endoscopically visible columnar-lined oesophagus >1cm with or without IM constitutes BO, whereas the American College of Gastroenterology requires IM within the columnar-lined epithelium as a pre-requisite for BO.^4^^,^^5^ Nevertheless, there is consensus that the presence of IM and the BO segment length is associated with higher cancer risk.^6^, ^7^, ^8^ Both the BSG and the new ACG guidelines now recommend that the presence of IM and length of BO segment should dictate surveillance intervals.^4^^,^^9^ The gold standard diagnostic tool for BO is gastroscopy with biopsies, and the length of BO can be estimated using the Prague criteria (Figure. 1).^10^

The Cytosponge is a minimally invasive cell collection device that samples the entire oesophagus and is coupled with a biomarker, Trefoil- factor 3 (TFF3) to detect IM (Figure. 1).^11^^,^^12^ The recent **B**arett's o**E**sophagus **S**creening **T**rial 3 (BEST3) showed that an offer of the Cytosponge can diagnose 10 times more BO than usual care, suggesting that it could be used for population-based screening for BO.^13^ Manual interpretation of Cytosponge results, however, is labour intensive but recent work has shown that a deep learning triage approach on digitized whole-slide images (WSIs) of TFF3 slides (Figure. 1a) performs the tasks of pathologists with high accuracy and can reduce pathologist workload by 57%.^14^ Although TFF3 have historically been interpreted in a binary fashion, there is significant variation between patients in the number of TFF3 positive gland groups seen in each slide. Extrapolating from biomarker use from other screening programmes, a quantitative biomarker threshold can be used to determine clinical management. For example, altering the quantitative threshold of faecal immunohistochemical test for colon cancer can be advantageous to optimise biomarker performance and reduce overdiagnosis.^15^ We, therefore, sought to understand whether the number of TFF3 positive gland groups can distinguish between pathologies with clinically relevant BO compared to shorter BO segment or IM of the gastro-oesophageal junction (GOJ), and further, whether TFF3 quantification can indicate the length of BO. Arguably those patients with a positive TFF3 result who have focal IM of the GOJ could be spared a confirmatory gastroscopy if this was predicted in advance.

The first aim of this study was to determine whether the TFF3 count generated by expert pathologists correlates with the presence of BO. The second aim was to use a machine learning approach on digitised TFF3 slides to quantify the extent of IM, correlate this with BO segment length and determine a threshold for predicting clinically relevant disease.

## Methods

### Cytosponge and Trefoil Factor 3

The Cytosponge comprises a compressed spherical sponge that is attached to a thread and enclosed within a dissolvable vegetarian capsule. Upon swallowing, the capsule dissolves after 5-7 minutes to release the sponge which is withdrawn by pulling on the thread where it collects cells from the proximal stomach and entire oesophagus.^11^ The sponge is then placed into preservative fluid and transported to the laboratory where it is centrifuged into a homogeneous clot and embedded into paraffin blocks. Sections of the paraffin blocks are then stained for haematoxylin and eosin (H&E), as well as immunohistochemistry for TFF3, a specific biomarker for IM.^12^ For the BEST2 and BEST3 trials, TFF3 staining was performed on slides 2 and 15 on serial sections according to our established protocol (proprietary monoclonal antibody; BD Diagnostics, Durham, North Carolina, USA) using standard protocols on a BOND-MAX autostainer (Leica Biosystems, Newcastle upon Tyne, UK).^16^ Slides 2 and 15 were selected based on previous Cytosponge studies which evaluated consecutive H&E slides and determined that a superficial and deep section around 12 slides apart was optimal.^12^^,^^17^

### Proof of concept using manual TFF3 gland count from the BEST3 study as a predictor of Barrett's oesophagus

The protocol and methodology for the BEST3 trial had been published previously.^13^^,^^18^ Briefly, the BEST3 trial (ISRCTN68382401) was a multicentre, pragmatic, randomised controlled trial conducted in 109 primary care practices in England between March 2017 to 2019.

In BEST3, TFF3 was reported in a binary fashion by specialist gastrointestinal pathologists. Pathologists also manually counted the number of TFF3-positive glands groups that were present in TFF3-positive WSIs (Figure 1c). These results were recorded but were not used to inform clinical decisions since all TFF3 positive patients were invited for a gastroscopy, and the results of the gland counts have not been published. There were 4 pathologists that were involved in counting TFF3 gland count, and each pathologist needed to be trained by our expert pathologist (M.O.D) and had to pass a test before being eligible to perform manual TFF3 counting.

For this study, using the TFF3 gland counts from slides 2 and 15, we subdivided the gland count into four different parameters to determine which parameter could best predict the diagnosis of BO: 1) TFF3 gland count from slide 2 only, 2) TFF3 gland count from slide 15 only, 3) highest gland count between slide 2 and 15, and 4) the average TFF3 gland count from slide 2 and 15. For BO, we used a cut-off of C*≥*1 or M*≥*3 and we defined these as clinically relevant BO and compared them to focal IM pathologies which we defined as having either short segment BO (C*<*1 and M*<*3) or no BO on gastroscopy, but given their TFF3 positivity, could have IM of the GOJ or gastric cardia.

### Ethics

Ethical approval was not required for this study. For the BEST3 trial, ethics approval, however, was previously given by the East of England-Cambridge East Ethics Committee, reference 16/EE/0546. For the BEST2 study, ethics approval was obtained from the East of England–Cambridge Central Research Ethics Committee, reference 10/H0308/71. Informed consent from all participants were obtained from the BEST2 and BEST3 studies.

### Statistical analysis

For the BEST3 study, participants were eligible for recruitment if: they were aged ≥50, had a history of heartburn indicated by a prescription of acid suppressant therapy for at least 6 months and had not undergone a gastroscopy within the preceding 5 years. Participants recruited into the study were randomised to usual care (control arm), or an offer of a Cytosponge test (intervention arm) and the primary outcome of the study was the number of BO diagnosed at 12 months after enrolment. All pathologists interpreting Cytosponge results were blinded to gastroscopy results. The BEST3 study was powered based on the expected proportion of BO that would be diagnosed over 12 months which was estimated to be 1.38% in the intervention group and 0.60% in the usual care group, and of which a sample size of 6764 patients was required to give a power of 90% at a significance of 5%. A subset of the data from the BEST3 trial was used for the current analysis.

**Figure 2:**
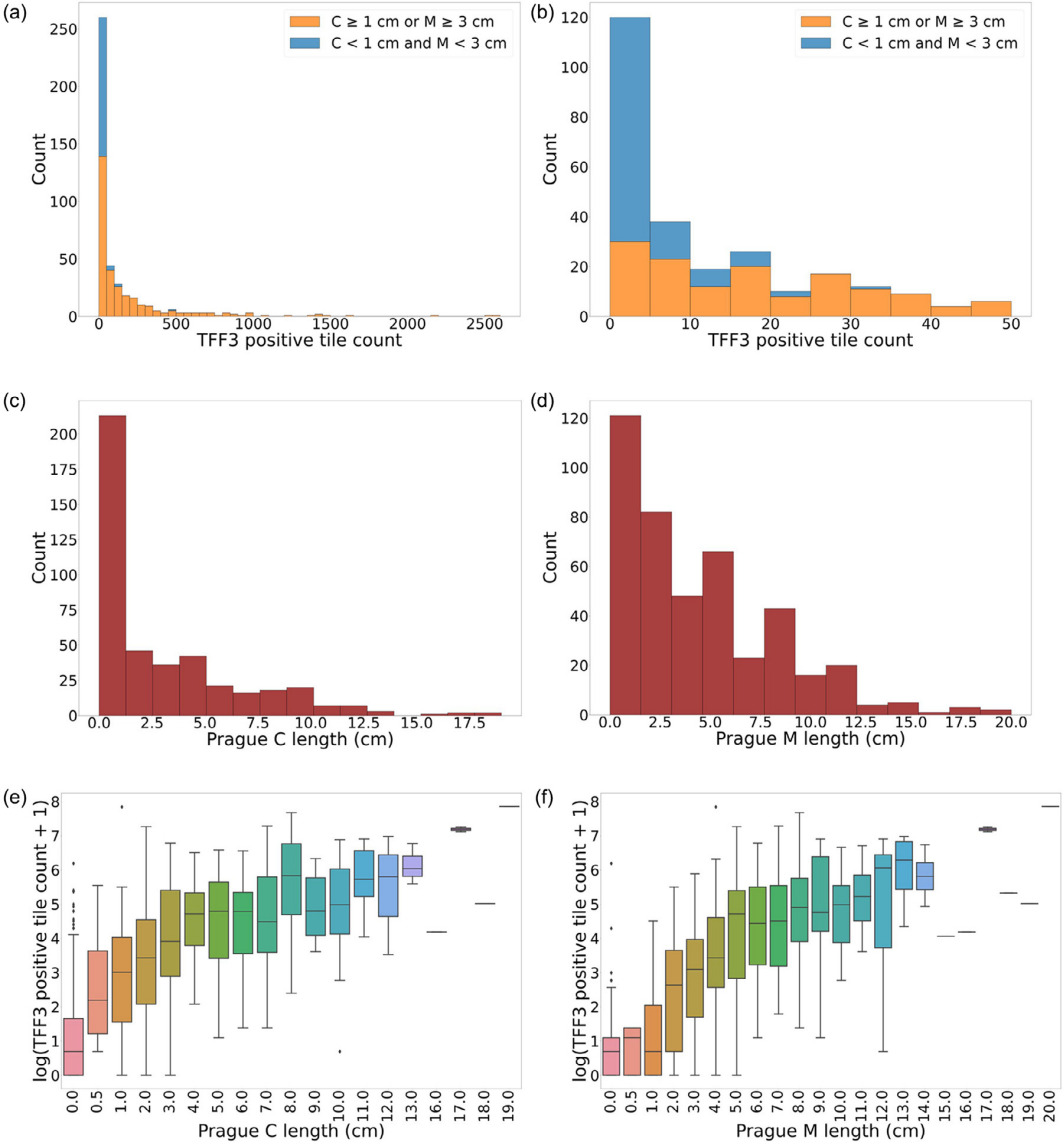
**Data distributions. (a)** A stacked-bar histogram of the machine-learning derived TFF3-positive tile counts of BEST2 patients (*n* = 529) who underwent the Cytosponge-TFF3 test. **(b)** Magnified view of the stacked-bar histogram from **(2a)** focusing on TFF3-positive tile count range of 0–50. **(c)** A histogram of the ground truth of C lengths of BEST2 patients. **(d)** A histogram of the ground truth of M lengths of the BEST2 patients. **(e)** A box-and-whisker plot showing the log of TFF3-positive tile count versus C length. **(f)** A box-and-whisker plot showing the log of TFF3-positive tile count versus M length. For figures a-d, patients with zero TFF3 positive tiles were excluded from the plots.

To develop our deep learning approach, we utilized patients from the BEST2 trial (*n* = 529). Briefly, the BEST2 (UK Clinical Research Network Study Portfolio 9461) trial is a case-control study that is conducted in secondary care which is still recruiting (2011 – present) and evaluated the performance of the Cytosponge among patients with a known diagnosis of BO compared with non-BO controls with reflux symptoms.^16^ For this study, no randomization was performed, but pathologist were blinded to the results of the endoscopy. Sample size was determined by assuming a sensitivity of 80% and specificity of 90% for TFF3 for diagnosing BO and we would have needed to recruit at least 600 BO and 450 controls to ensure a 95% CI of approximately ±3%. However, as the aim of the current manuscript differs to that of the original BEST2 study, only a subset of the data from the BEST2 was used for the current analysis. For TFF3 quantification, we used a deep learning pipeline we previously developed which classifies regions of WSI of TFF3-stained Cytosponge as TFF3-positive or TFF3-negative with a precision (positive predictive value) of 0.903 and recall (sensitivity) of 0.919.^14^ The deep learning model was a convolutional neural network, a model well-suited to taking image data as an input parameter.^19^ The images used to train the model were small square 400 × 400 pixel or 200 × 200 µm sub-regions of larger WSI, known as “tiles.” These tiles are sampled from digital annotations of the WSI demarcating where regions of TFF3-positivity lie. Tile sampling is performed by putting a bounding box around each digital annotation and extracting as many non-overlapping tiles from that box as possible. Only tiles with at least 66% of their area overlapping with the digital TFF3 annotation were used. This process generates a training dataset of tiles used to teach the model what TFF3-positivity looks like so that the model can then be applied to new examples for validation. An existing software library, published as a preprint, was used to perform the tiling task.^20^ Once trained on these tiles, this deep learning model was applied to the WSIs used for this paper to generate the automated TFF3-positive tile counts (Figure 2) used to predict segment length in this paper.

The width of tiles is an important parameter when training these kinds of models, as it determines the field of view made available to the machine learning model. We used square tiles with a 400-pixel edge length.^14^ Since the slides were scanned at 40x resolution, this translates to square images with a 100-micron edge length. This was chosen as it is roughly the size of one-fourth of an average columnar gland; at this scale, one or two TFF3-positive goblet cells tend to be very clearly visible and distinguishable by a human pathologist (Figure 1). Cytosponge samples showing no evidence of gastric cardia mucosa on H&E, suggesting that the sponge may not have adequately sampled from the distal oesophagus, were excluded. This is a standard quality control metric used in Cytosponge slide evaluation.

For descriptive statistics, the mean and standard deviation of the TFF3 gland count between patients with and without BO were calculated. Equality of variance was tested using Levene's test. Welch T-test was used to compare means between groups. We performed univariate logistic regression as well as plotted the area under the receiver operating characteristic curve (AURoC) for each TFF3 gland count parameter to determine which parameter best predict a diagnosis of clinically relevant BO (C*≥*1 or M*≥*3) versus focal IM pathologies (BO C*<*1 and M*<*3 or IM of the GOJ). For the machine learning model, logistic regression was used to predict segment length from the IM tile count. Spearman's correlation was performed to assess the relationship between IM tile counts and BO segment length. Analyses were performed using R version 4.1.3. Class-balanced accuracy was computed using R's Yardstick library and is defined as the mean of sensitivity (also known as recall or true positive rate) and specificity (also known as selectivity or true negative rate) for the clinically-relevant segment length class; this is preferred to simple accuracy because there is an imbalance in the number of ground truth clinically relevant segment length patients to non-clinically relevant patients. Determination of model decision boundaries (TFF3-positive tile count cutoffs identified by the model above which the model then predicts that a patient has clinically relevant BO) was performed with R's MASS library.^21^

### Role of funding source

BEST2 was funded by Cancer Research UK (CRUK; 12088 and 16893). The BEST3 trial was funded by CRUK, National Institute for Health Research (NIHR) and Medical Research Council. Both studies received support from Addenbrookes Human Tissue Bank which is supported by the Cambridge Biomedical Research Centre (BRC-1215-20014) and the Experimental Cancer Medicine Centre. A.G.B, F.M and R.C.F had access to the data and take responsibility for the integrity of the data and the accuracy of data analysis. F.M and R.C.F had the final responsibility to submit for publication. Both funding bodies had no role in the study design, data analyses, interpretation or writing of the manuscript.

## Results

### Association between manual TFF3 gland group counts and a diagnosis of Barrett's oesophagus

The first aim was to determine whether the manual TFF3 counts obtained from the Cytosponge correlated with the diagnosis of clinically relevant BO or focal IM pathologies. In the BEST3 trial, 1654 patients swallowed the Cytosponge and 231 were positive, for which 221 underwent gastroscopy. For this analysis, after omitting those with missing BO length and TFF3 gland count data, 52 patients had a clinically relevant BO (C*≥*1 or M*≥*3) and 146 patients had focal IM pathologies (short BO C<1 and M<3, or IM of the GOJ). We found that for all the TFF3 parameters: 1) TFF3 gland count from slide 2 only, 2) TFF3 gland count from slide 15 only, 3) the average TFF3 gland count from slides 2 and 15, and 4) highest gland count between slide 2 and 15, on univariate analysis; there was a significant difference in the TFF3 gland count between patients who had clinically relevant BO compared to those with focal IM pathologies (Table 1).

**Table 1:** Comparison of different TFF3 gland count parameters from the Cytosponge and diagnosis of BO.

Gland Count Parameter	Pathology			
	Clinically relevant BO$ (*n* = 52)	Focal IM Pathologies& (*n* = 146)	Mean Difference (95% CI)	p-value*
Gland count from slide 2, mean (SD)	6.71 (4.44)	2.86 (3.09)	3.86 (2.53 – 5.19)	*<*0.001
Gland count from slide 15, mean (SD)	7.58 (5.04)	3.15 (3.05)	4.43 (2.94 – 5.91)	*<*0.001
Average gland count between slide 2 and 15, mean (SD)	7.14 (4.66)	3.00 (2.93)	4.14 (2.76 – 5.52)	*<*0.001
Highest gland count between slide 2 or 15, mean (SD)	7.73 (4.88)	3.66 (5.86)	4.07 (2.63 – 5.51)	*<*0.001

BO, Barrett's oesophagus; TFF3, Trefoil-factor 3; IM, intestinal metaplasia; SD, standard deviation; CI, confidence interval.

$ Clinically relevant BO refers to BO C*≥*1 or M*≥*3.

& Focal IM pathologies refer to BO C*<*1 and M*<*3 or IM of the GOJ.

⁎ Welch T-test used due to inequality of variance.

We then performed univariate logistic regression and calculated the AURoC separately for each parameter to determine which parameters best predict the presence or absence of clinically relevant BO. Our results showed that the AURoC of all TFF3 gland count parameters were equally predictive of having clinically relevant BO (AURoC for all parameters ranged from 77.4% to 78.4%), but TFF3 gland count from slide 15 alone (AURoC 78.4%, 95% CI 71.1-85.8%), and the average TFF3 gland count between slides 2 and 15 (AURoC 78.4%, 95% CI 71.1-85.7%) were equally good predictors of having a diagnosis of clinically relevant BO (Supplementary Table 1 and Supplementary Figure 2). However, given that the average gland count between slides 2 and 15 provides a better representation for the whole Cytosponge sample where slides 2 and 15 are taken at different depth (slide 2 being more superficial and slide 15 deeper into the paraffin block) compared to taking slide 15 alone, this parameter was hence selected. Having observed these findings, we proceeded to investigate whether this relationship could be replicated, and improved upon, using a machine learning model instead of manually counted TFF3-positive gland groups.

### Correlations between automated intestinal metaplasia tile count and BO segment length

We used the BEST2 trial cohort (*n* = 529) to derive our automated prediction model since this study had a range of segment lengths in patients who were undergoing BO surveillance. We took the raw output from the deep learning-based Cytosponge-TFF3 IM-prediction model^14^, and extracted the positive tile count from within the WSI.^22^ Interpretation of TFF3 using this model had been previously performed in a binary fashion – TFF3 positive or negative. We used the number of TFF3-positive tiles as a measure to quantify IM. As patients within the BEST2 study underwent a gastroscopy on the same day as the Cytosponge, we then correlated TFF3-positive tile counts with the BO segment length measured at gastroscopy (Figure 2a–d). Our results showed that the quantity of TFF3-positive tiles correlated with segment length with a Spearman's rank correlation coefficient of 0*.*73 for C length and 0*.*77 for M length (Figure 2e–f).

As a negative control, we also checked whether there was a correlation between the number of tiles identified as gastric cardia and BO lengths. Specifically, Cytosponge samples columnar epithelial from the gastric cardia and these are used as quality control for adequate sampling. It is expected that there should be no correlation between the gastric tile counts and BO length containing TFF3 positive IM. Indeed, for the BEST2 cohort, Spearman's rank correlation coefficients between the gastric tile counts and the C length was *−*0*.*024, and for M length was *−*0*.*046 confirming the validity of our approach.

### Exploration of a classification-based predictive model to distinguish clinically relevant BO disease from focal IM pathologies

We next asked if the significant correlation between our machine learning approach, which automatically identified TFF3 positive tiles, could be leveraged in an optimized prediction model to differentiate clinically relevant BO from focal IM pathologies. After training the model on our BEST2 cohort, we performed a 5-fold cross-validation which showed that the mean class-balanced validation accuracy of the logistic regression was 0.84 (95% CI 0.77-0.90), the mean validation precision of the diagnostically positive class was 0.95 (95% CI 0.87-1.00), the recall 0.74 (95% CI 0.62-0.83), and the F1 score (the harmonic mean of precision and recall) was 0.83 (95% CI 0.74-0.90). Class balanced accuracy is an accuracy measurement meant to be robust for imbalanced class sizes (in this context, the sizes of the clinically relevant BO and focal IM pathologies patient groups); it is the average of the recalls achieved for each class. We also determined the decision boundary or threshold for the minimum number of machine-learning identified TFF3-positive tiles required for the model to consider a patient to have clinically relevant BO (C≥1 or M≥3). This was 16.6 tiles, taking the mean across the validation folds (Table 2).

**Table 2:** Model performance and results on the BEST2 validation cohort.

Fold	Accuracy	Balanced accuracy	Precision (C)	Precision (F)	Recall (C)	Recall (F)	F1 (C)	F1 (F)	Tile threshold
**1**	0.86	0.87	0.96	0.75	0.80	0.95	0.87	0.84	15.0
**2**	0.82	0.83	0.91	0.75	0.74	0.92	0.82	0.83	15.6
**3**	0.80	0.84	0.96	0.63	0.73	0.94	0.83	0.76	19.3
**4**	0.78	0.82	0.98	0.65	0.66	0.98	0.79	0.78	17.7
**5**	0.84	0.85	0.94	0.76	0.76	0.94	0.84	0.84	15.5
**Mean**	0.82	0.84	0.95	0.71	0.74	0.94	0.83	0.81	16.6
**SD**	0.030	0.022	0.026	0.061	0.052	0.021	0.033	0.037	1.82

C, clinically relevant BO (C≥1 or M≥3); F, focal IM pathologies (BO C<1 and M<3 or IM of the GOJ); SD, standard deviation.

F1 score refers to the weighted average of precision and recall. Tile threshold denotes the cut-off above which the model considers a patient to have clinically relevant BO.

### Classification model performance and validation on the BEST3 cohort

After training our model, we then re-tested it on patients in the BEST3 trial who had a TFF3-positive Cytosponge result. For this analysis, we restricted our analysis only to patients who had a recorded BO length (*n* = 158). Applying the model trained on the BEST2 cohort to the BEST3 cohort, we observed a precision of 0.91 (95% CI 0.85-0.97) for focal IM pathologies (78/86) with a class-balanced accuracy of 0.77 (95% CI 0.69-0.84) (Table 3). Crucially, by performing a gastroscopy on the 45% (71/158) patients our model predicted to have a clinically relevant BO, it would allow for the remaining 55% (87/158) of test set patients to avoid gastroscopy, for which we would only have missed 5.1% of patients with BO segment of C≥1 or M≥3 (8 false negatives of 158), and they presumably have focal IM within their segment. Applying our model to the BEST3 cohort also revealed that the decision boundary of the minimum number of TFF3-positive tiles required for a diagnosis of long-segment BO is 16.7, which we rounded down to a discrete 16 tiles. This number is identical to the value we obtained as a mean of the decision boundaries of our cross-validation fold (Table 3).

**Table 3:** Results of training a logistic regression model on BEST2 patients without cross-validation and then inferring on BEST3 patients. Shown are model results when applied on the BEST3 cohort.

Accuracy	Balanced accuracy	Precision (C)	Precision (F)	Recall (C)	Recall (F)	F1 (C)	F1 (F)	Tile threshold
0.75	0.77	0.55	0.91	0.83	0.71	0.66	0.80	16.7

C, clinically relevant BO (C≥1 or M≥3); F, focal IM pathologies (BO C<1 and M<3 or IM of the GOJ).

F1 score refers to the harmonic mean of precision and recall. Tile threshold denotes the cut-off above which the model considers a patient to have clinically relevant BO.

## Discussion

Our results show a correlation between the number of TFF3-positive tiles, i.e IM quantification, and BO diagnosis. Furthermore, the number of TFF3-positive tiles allowed us to predict with high accuracy patients harbouring clinically relevant BO from focal IM pathologies that likely have a low cancer risk. This establishes TFF3-positive tile counts as a relevant new biomarker for consideration in the management of BO.

First, quantification of IM on Cytosponge specimens could be important, particularly if the Cytosponge is to be used for population-based screening for BO or OAC. The concept of Cytosponge screening is as a triage for gastroscopy but this could burden endoscopic services unless thresholds for referral are optimised. In screening, it is important to reduce overdiagnosis and this has been very topical when considering the imperative to improve early diagnosis on one hand, but to ensure this is not burdensome to the individual or the healthcare system.^23^

Secondly, a longer BO segment has a higher cancer progression risk. A recent study that utilised clinical risk factors to develop a model to predict BO progression showed that BO length (per 1cm increase) is a significant predictor of progression (HR 1.12, 95% CI 1.08-1.18)^24^. The length criteria is also incorporated in the BSG guidelines to dictate surveillance intervals for gastroscopy whereby patients with maximum segment length *≥*3cm should undertake gastroscopy every 2-3 years, and every 3-5 years for those *<*3cm.^4^ The higher cancer risk among those with a longer BO segment is because IM is distributed unevenly within the BO segment and biopsies only sample a tiny proportion of the entire BO segment and are prone to sampling error.^25^^,^^26^ There is an argument that screening for TFF3 as a surrogate for IM may be limited by the reduction in goblet cells as BO progresses to cancer, however, our BEST2 trial showed that the sensitivity for TFF3 in the presence of dysplasia is not reduced by dysplastic grade.^27^ The advantage of the Cytosponge is that it could sample a larger area of the oesophagus compared to conventional biopsies and that no material is loss and the Cytosponge parrafin blocks are stained for additional biomarkers (atypia and p53) for assessment of dysplasia.

IM limited to the GOJ is controversial and previously thought to harbour little malignant potential, although some experts currently believe that it is clinically important and could give rise to OAC and is under-diagnosed.^28^ Nevertheless, however, current societal guidelines have not recommended routine biopsies of the GOJ, and even if it is diagnosed, surveillance or treatment is not recommended.

Thirdly, in our trials to date, TFF3 assessment has been used as a binary biomarker in which only a single positive cell is required to be considered positive.^11^^,^^13^^,^^16^ However, we hypothesised that a quantitative TFF3 assessment could reduce overdiagnosis of focal IM pathologies including short segments BO that is likely to be inconsequential. Since we have previously developed a machine learning framework, we were keen to extend this so that the analysis could be automated. The previous algorithm assessed whether the specimen contained TFF3 and provided a confidence estimate that was able to reduce pathologists' workload by 57% but still matched the diagnostic performance of the pathologists.^14^ However, in that study, TFF3 results were reported as binary only.

Here, we refined our machine learning algorithm further. We trained our model on patients with BO within the BEST2 trial, where we compared BO segment length of C*≥*1 or M*≥*3 (long segment BO) to focal IM pathologies (short segment BO of C*<*1 and M*<*3, or those with likely IM of the GOJ or gastric cardia). We selected the BEST2 over the BEST3 study to develop our machine learning algorithm because the BEST2 was a case-control study of patients with a known BO diagnosis who received a Cytosponge, followed by gastroscopy whilst the BEST3 trial was a population-based screening study among patients with reflux symptoms who were taking acid-suppressant therapy. Hence, BEST2 was biased towards patients with longer segment BO, and this was confirmed by plotting the kernel density estimation plots on the BO length (Supplementary Figure 1a and b). Indeed, 59.5% of patients in the BEST2 study had segment length C*≥*1 or M*≥*3 compared to 29.6% in BEST3. We note that developing our model on the BEST2 cohort with a longer segment length and validating our model on the BEST3 study among patients with focal IM pathologies, could lead to poor calibration or overestimation of our results. However, this was not seen in our study, in which the number of TFF3-positive tile counts used to predict clinically relevant BO was almost identical at 16.6 and 16.7 tiles, respectively, without having to re-calibrate for the shorter BO segment length in the BEST3 population. Our results could have significant clinical implications given that the Cytosponge would need to be applied to the general population as a pro-active screening tool for BO and OAC. In a hypothetical screening population, patients who are offered the Cytosponge for screening could have their Cytosponge results interpreted through an automated machine learning approach, and if a patients’ TFF3 quantification is above our threshold, it would then trigger a gastroscopy. Those below the cut-off who likely harbour focal IM which could constitute short BO or IM of the GOJ which harbour lower risk of malignant potential^29^ and could be followed-up with a repeat Cytosponge in 2-3 years, although this suggested follow-up interval is based on expert opinion only whilst more data is awaited. This strategy would allow prioritization of patients with clinically relevant long BO and avoid overburdening endoscopic services with shorter and less clinically important pathologies. It should be noted that if the Cytosponge test is used as a diagnostic tool for patients with reflux symptoms, it is likely that any patient with TFF3 positive finding would be referred for endoscopy, since symptomatic referral would lead to a more cautious approach compard with proactive screening in patients who have not sought medical help.

This study has strengths and limitations. First, the training of the model was performed on a relatively large cohort of patients and the validation was done on an independent test set (BEST3), which was representative of a screening population for which the Cytosponge could be applied. However, the independent test set was relatively small, and efforts are now focusing on validating this model on a larger number of patients within the DELTA trial (ISRCTN91655550). Secondly, those who were positive for TFF3 but who did not have BO on gastroscopy were presumed to have IM of the GOJ and hence, were labelled under the category of ‘focal IM pathologies’. The diagnosis of IM of the GOJ however, was not always confirmed given that we did not perform routine biopsies of the GOJ. However, since TFF3 is specific for IM, it is quite likely that the Cytosponge had picked up inconspicuous IM somewhere along the upper gastrointestinal tract, and in the absence of BO, the most likely source of IM is from the GOJ.

In conclusion, this study suggests that a Cytosponge-based screening strategy could accurately predict patients harbouring clinically relevant BO to prioritise endoscopic services and could avoid or delay gastroscopy among those predicted to have focal IM pathologies. This strategy not only makes a Cytosponge-based screening programme feasible, but could also improve the cost-effectiveness associated with screening for BO or OAC.

## Contributors

Conceived and designed the study: R.C.F. Led the analysis: A.G.B. Contributed to the analysis: W.K.T. Histological Interpretation: M.O'D. Wrote and critically reviewed the manuscript: A.G.B, W.K.T, M.O'D., R.C.F and F.M. All authors had access to the data in the study and accept responsibility to submit for publication. The final manuscript was approved by all authors.

## Data sharing statement

Deidentified participant data from the BEST2 and BEST3 studies as well as documented code with instructions for running the analyses shown in this paper, including generating diagnostic plots for the regression model, can be found at the following public repository: https://github.com/markowetzlab/barretts-segment-length-predictor. The study protocol for the BEST2 and BEST3 study have been made publically available along with the publication of the manuscript.

## Declaration of interests

R.C.F. and M.O'D. are named on patents related to the Cytosponge-TFF3 which has now been licensed by the Medical Research Council to Covidien GI Solutions (now Medtronic). The Cytosponge-TFF3 device has been CE marked and is cleared by the US Food and Drug Administration. R.C.F. is co-founder and shareholder in Cyted, a company working on early detection technology. M.O'D. is also a co-founder and shareholder of Cyted and is a part-time employee as the lead Cytosponge pathologist for Cyted. FM is a founder, director, and shareholder of Tailor Bio and has received consulting fees from the Alan Turing Institute and is also a member of the expert advisory groups for the Turing-Roche partnership. F.M. also received consulting fees as part of the CRUK expert panel. F.M. is also a SAB member of the EPSRC Centre for Mathematical imaging in Healthcare, Sonderforschungsbereich Jak-Stat Vienna and CRUK Early Diagnosis Consortium. The other authors declare no conflict of interest.

## Appendix Supplementary materials

Image, image 1

Image, image 2

Image, image 3

Image, application 4

Image, application 5
