# Supplementary material for: Quantification of TFF3 expression from a non-endoscopic device predicts clinically relevant Barrett's oesophagus by machine learning
Source: eBioMedicine. 2022 Jul 15;82:104160. doi: 10.1016/j.ebiom.2022.104160 (PMC9297109; doi:10.1016/j.ebiom.2022.104160)
Supplement: Supplementary file 1 [file mmc1.docx]

**Supplementary Material**

**Supplementary Table 1**

**Figure legend**

**Supplementary Figure 1**. BEST2 vs. BEST3 segment length distributions **(a)** Kernel density estimation plots of the Prague C lengths of BEST2 and BEST3 patients who underwent the Cytosponge-TFF3 test. **(b)** Kernel density estimation plots of the Prague M lengths of BEST2 and BEST3 patients who underwent the Cytosponge-TFF3 test. Patients with zero TFF3-positive tiles were excluded from both plots.

**Supplementary Figure 2.** Area under the receiver operating curve (AUC) for all 4 TFF3 gland count parameter showed almost identical performance with slide 15 alone and average of slide 2 and 15 being identical. True positive percentage represents the sensitivity and false positive percentage represents the 1-specificity.
