# Supplementary figures and images for: Quantification of TFF3 expression from a non-endoscopic device predicts clinically relevant Barrett's oesophagus by machine learning
Source: eBioMedicine. 2022 Jul 15;82:104160. doi: 10.1016/j.ebiom.2022.104160 (PMC9297109; doi:10.1016/j.ebiom.2022.104160)

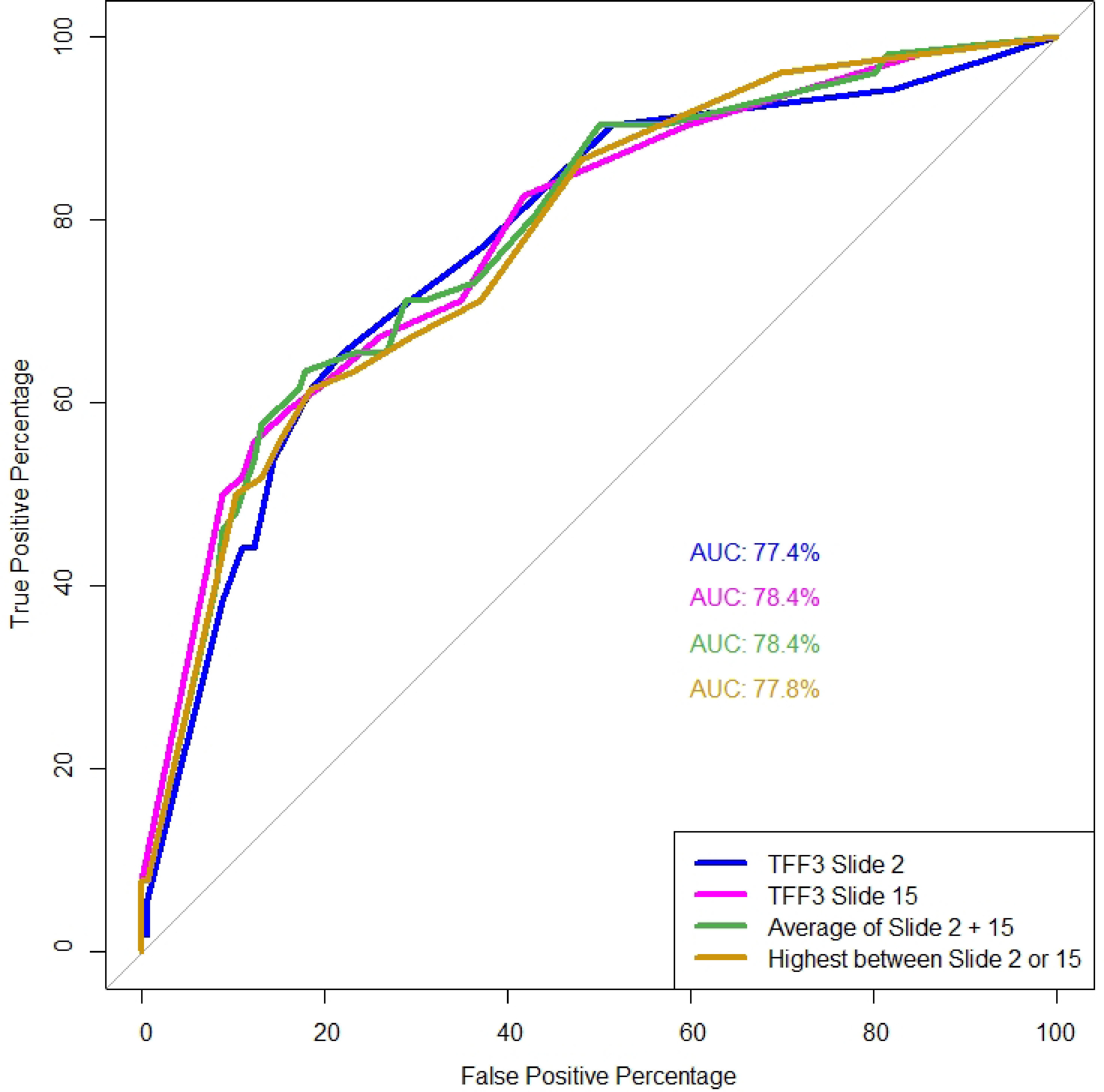

Supplement: Supplementary file 2 [file mmc2.jpg]

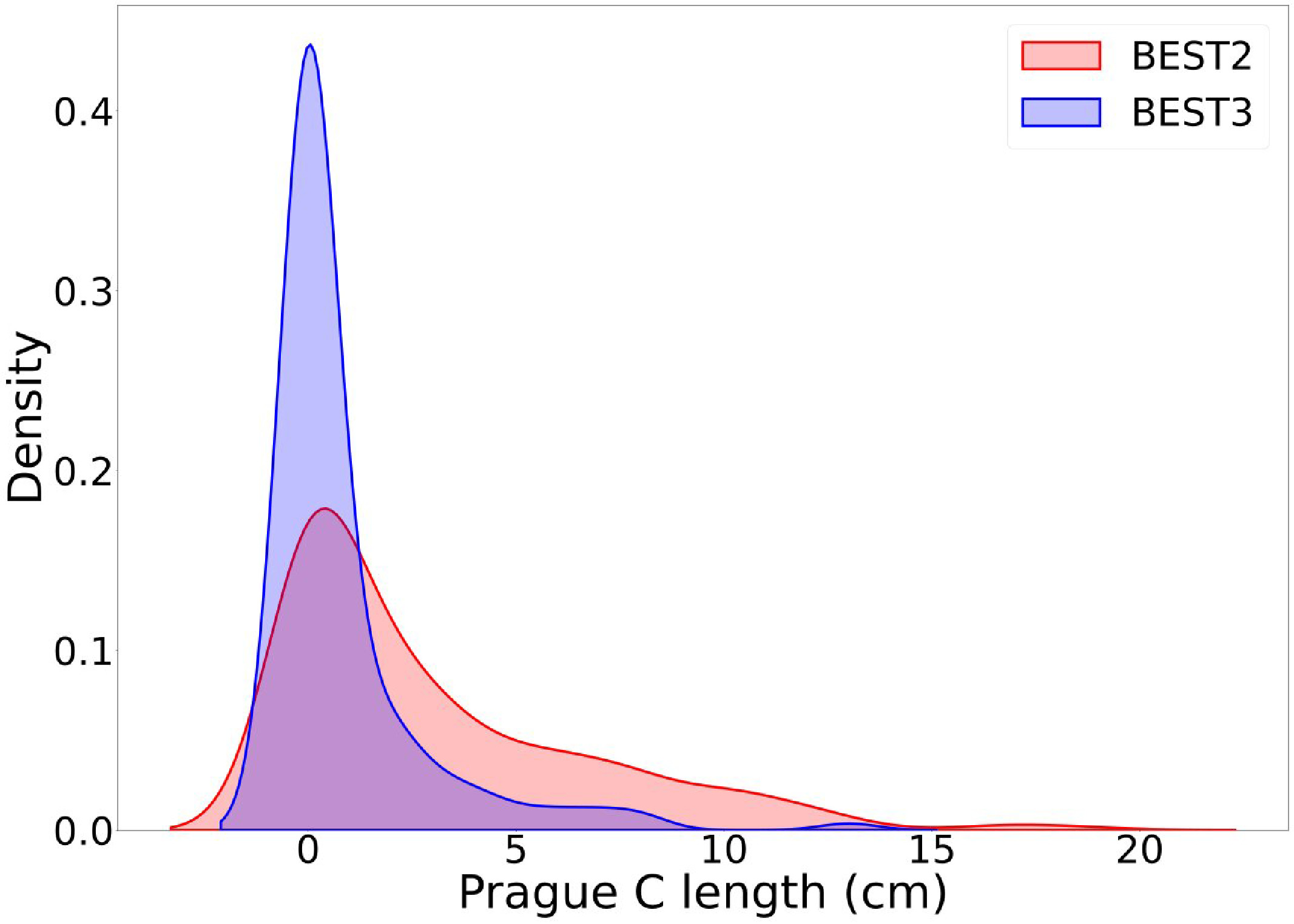

Supplement: Supplementary file 3 [file mmc3.jpg]

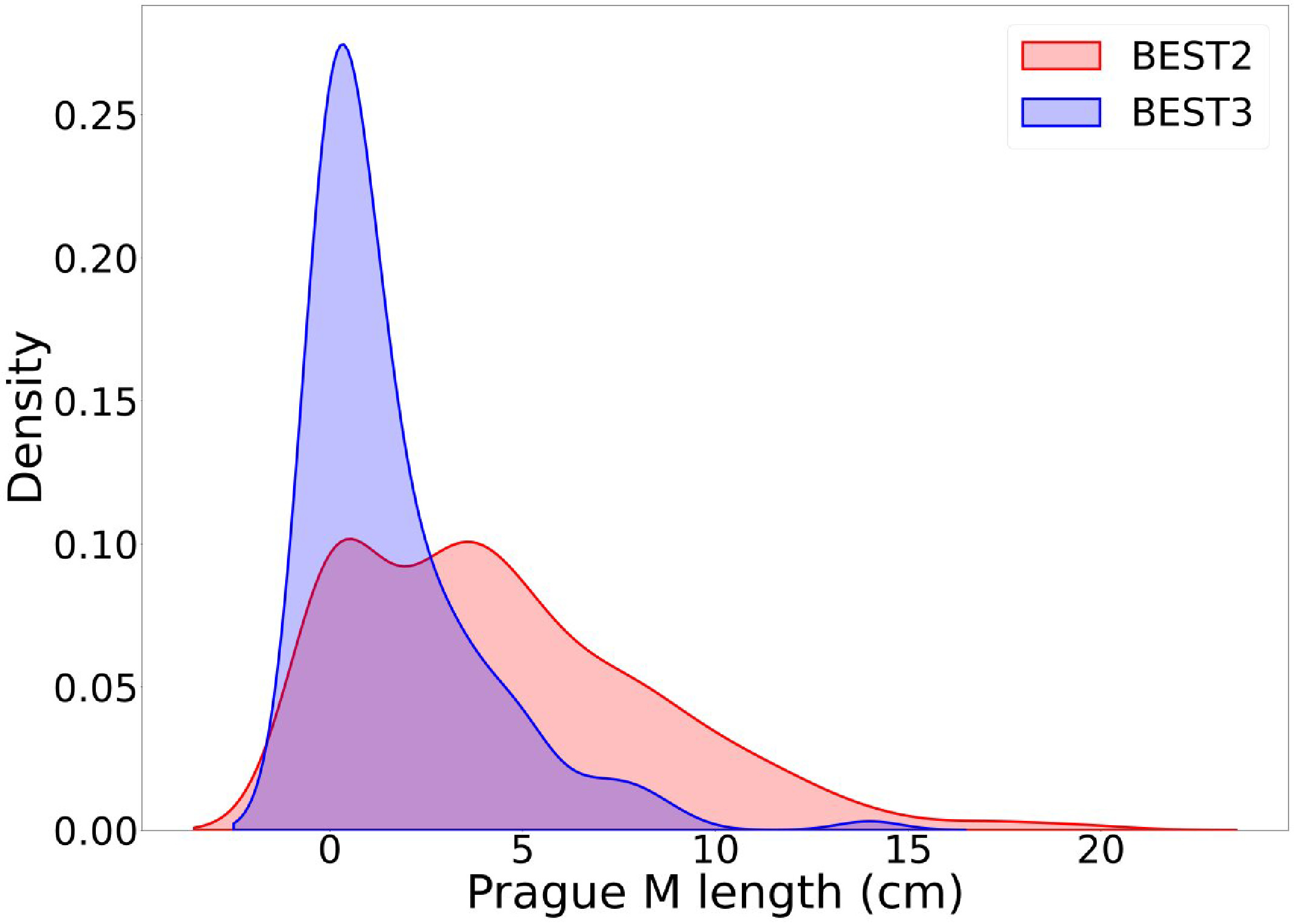

Supplement: Supplementary file 4 [file mmc4.jpg]
