# Supplementary material for: Quantification of TFF3 expression from a non-endoscopic device predicts clinically relevant Barrett's oesophagus by machine learning
Source: eBioMedicine. 2022 Jul 15;82:104160. doi: 10.1016/j.ebiom.2022.104160 (PMC9297109; doi:10.1016/j.ebiom.2022.104160)
Supplement: Supplementary file 5 [file mmc5.docx]

**Supplementary Table 1.**

| Table 1. TFF3 gland count parameter for predicting clinically relevant BO | | |
| --- | --- | --- |
| TFF3 Gland Count Parameter | **Odds ratio (95% CI)** | **AURoC, % (95% CI)** |
| TFF3 Slide 2 | 1.311 (1.193 – 1.442) | 77.4 (69.8-84.9) |
| TFF3 Slide 15 | 1.336 (1.213– 1.472) | 78.4 (71.1-85.8) |
| Average of slide 2 + 15 | 1.344 (1.219-1.483) | 78.4 (71.1-85.7) |
| Highest between Slide 2 or 15 | 1.324 (1.201 – 1.461) | 77.8 (70.6-85.0) |

BO, Barrett’s oesophagus; AURoC, Area under receiver operating curve; CI, confidence interval

Clinically relevant BO refers to BO C≥1, or M≥3
